# Advances in Understanding of Desiccation Tolerance of Lichens and Lichen-Forming Algae

**DOI:** 10.3390/plants10040807

**Published:** 2021-04-20

**Authors:** Francisco Gasulla, Eva M del Campo, Leonardo M. Casano, Alfredo Guéra

**Affiliations:** Department of Life Sciences, Universidad de Alcalá, Alcalá de Henares, 28802 Madrid, Spain; eva.campo@uah.es (E.M.d.C.); leonardo.casano@uah.es (L.M.C.)

**Keywords:** desiccation tolerance, lichen, phycobionts, poikilohydric, stress response

## Abstract

Lichens are symbiotic associations (holobionts) established between fungi (mycobionts) and certain groups of cyanobacteria or unicellular green algae (photobionts). This symbiotic association has been essential in the colonization of terrestrial dry habitats. Lichens possess key mechanisms involved in desiccation tolerance (DT) that are constitutively present such as high amounts of polyols, LEA proteins, HSPs, a powerful antioxidant system, thylakoidal oligogalactolipids, etc. This strategy allows them to be always ready to survive drastic changes in their water content. However, several studies indicate that at least some protective mechanisms require a minimal time to be induced, such as the induction of the antioxidant system, the activation of non-photochemical quenching including the de-epoxidation of violaxanthin to zeaxanthin, lipid membrane remodeling, changes in the proportions of polyols, ultrastructural changes, marked polysaccharide remodeling of the cell wall, etc. Although DT in lichens is achieved mainly through constitutive mechanisms, the induction of protection mechanisms might allow them to face desiccation stress in a better condition. The proportion and relevance of constitutive and inducible DT mechanisms seem to be related to the ecology at which lichens are adapted to.

## 1. Introduction

Water is the most abundant compound in any living being; water is the universal solvent where inorganic salts and biomolecules are dissolved in; water is essential for many important chemical reactions in organisms; water maintains the structure of cell macromolecules and membranes. However, there are some organisms that can survive the complete loss of their water content (<0.1 g H_2_O g^−1^ dry mass). When dried, desiccation-tolerant organisms enter a latent state, so-called anhydrobiosis, and stop any metabolic activity until the following rehydration. Desiccation-tolerance (DT) is a polyphyletic trait that can be found in a wide range of life forms, from bacteria to animals. In the Plant Kingdom, DT is frequent in algae and bryophytes, but rare in tracheophytes, only about 350 species of flowering plants and ferns are able to return to life after desiccation [1]. Classically, desiccation-tolerant plants have been divided in two groups, modified and fully desiccation-tolerant plants [2]. In the first group we can find vascular plants that only survive if desiccation occurs slowly (in days to weeks) and need 24 h or even more to recover their normal activity. These plants need time to shift from an active vegetative growth to a complete metabolic stop (anhydrobiosis) [3,4]. The fully desiccation-tolerant plants group is comprised by poikilohydric plants-like mosses or microalgae- that can survive very fast dehydration (less than 1 h) and are able to recover their normal metabolism within minutes upon rehydration. Here, tolerance seems to be primarily based on constitutive mechanisms and the ability to repair damage caused by desiccation [2,5]. Lichens belong to this last group, they do not have active mechanisms to control their water content and can resist fast dehydration.

Lichens are stable mutualistic symbioses between a fungus (mycobiont) and at least one photosynthetic partner (photobiont) that can be an eukaryotic algae (phycobiont) and/or a cyanobacteria (cyanobiont). In this association, the mycobiont is the exhabitant that forms a thallus around the photobiont, which provides carbohydrates to the fungus [6]. The oldest cyanobacterial and green algal lichens with heteromerous thallus anatomy (predominance of fungal cells over photobiont cells) so far found, are two fossils from the Lower Devonian (Lochkovian, approx. 415 Myr) [7]. These fossils show the characteristic thallus organization of extant foliose Lecanoromycetes lichens formed by an upper thin and compacted fungal hyphae layer (cortex), a photobiont layer and a lower loosely interwoven hyphae layer (medulla). The morphology and size of the cyanobiont and the phycobiont very strongly resemble extant *Nostoc* ssp. and *Trebouxia* ssp., respectively [7]. Recent time-calibration of ascomycete fungi and algal lichen-associated phylogenies show broad congruence and demonstrate that lichen symbiosis appeared during the Devonian period [8]. The origin of embryophytes is estimated in the middle Cambrian-Early Ordovician (∼515.2 Myr to 473.5 Myr) followed by a high diversification in the Silurian period (443.8 Myr to 419.2 Myr) [9]. Thus, lichens emerged in a land already conquered by bryophytes and vascular plants and had to adapt to niches unsuitable for the latter. Extant lichens have a very low growth rate, from millimetres to centimetres per year, and cannot compete directly against land plants for vital sources such as water, light or space. Lichens occupy habitats with low water retention capacity such as rock surfaces, sand, gypsum soils or tree bark, where land plants cannot survive. This adaptive strategy is mainly based on the tolerance to desiccation. Lichens are poikilohydric organisms, which means they do not have active mechanisms to regulate their water content, and therefore hydration fluctuates with the availability of water in the surrounding environment. When a lichen thallus is hydrated, the photobiont produces carbohydrates through the photosynthetic process-obviously in presence of light-, which are used in its own growth but also to feed the fungus partner. When lichens completely lose their water, they enter anhydrobiosis until the following rehydration. Theoretically, lichens can survive infinite cycles of desiccation/rehydration, the only requisite is to be hydrated -in the light- for enough time to compensate the energetic cost of fungus respiration and to make new carbohydrates for growth. In this sense, besides precipitation, lichens can utilize other water sources, such as fog and dew, and indeed have the ability to extract some moisture from the air, which can help to extend the hydration time. In addition, desiccation confers an extraordinary resistance to extremely high and low temperatures, to insolation and to UV irradiation [10], which allow lichens to conquer stressful environments such as hot deserts and high mountains. The strong similarity between the thallus organization and the photobiont taxonomy of Devonian and extant lichens suggests that early lichens also based their adaption strategy on poikylohydria and DT. This strategy had a great evolutionary success and earliest lichens diversified on land even after the rise and spread of multicellular plants. Nowadays this diverse group is found in almost all terrestrial habitats from the tropics to polar regions and dominate about ~7% of the earth’s terrestrial surface [11].

The scientific study of the specific physiological traits of lichens began in the 19th Century and demonstrated that lichens have the extraordinary capacity to tolerate desiccation for several months [12]. During the following century, lichenologists and other scientists tried to establish the limits and the ecophysiological significance of lichen DT. Probably, the major problem in studies carried out on survival of lichens under extreme conditions is to determine their viability. As we stated above, the growth rate of lichens is very slow, and the effects caused by moderate stress generally cannot be measured within a short period. Thus, “visual investigation of the viability of lichens is unreliable” [13]. Because of these difficulties, many kinds of physiological measurements and observational approaches were used to determine the ability of lichens to survive to extreme environments. One of these methods was to observe whether the symbionts isolated from a stressed lichen thallus grew in culture [14,15]. The measurement of CO_2_ exchange was also proven to be a reliable indicator for the response of lichens to any environmental influence. Jumelle [16] was the first to study the response of a lichen thallus to stress by using this technique. After him, several authors described different methods of measurement of CO_2_ exchange in a lichen thallus, reviewed in Kappen et al. [12]. At the end of the 20th Century began the massive use of chlorophyll fluorescence measurements to determine lichen fitness through the analysis of the photobiont photosynthesis performance [17]. The general conclusions of these studies were that the great majority of lichens are highly desiccation tolerant, but they do not survive embedded in water for a few days. Dry thalli can resist heat, up to 60 °C, however, a hydrated thallus dies when the temperature exceeds 35 °C. Lichens can also tolerate extremely low temperatures (−196 °C) when dry and when hydrated if cooling is slow enough.

During the end of the 20th Century and beginning of the 21st, scientists focused on understanding the physiological processes that lie behind lichen tolerance to environmental stress. Most of the studies addressed the physiological responses to desiccation-induced oxidative stress. The intracellular overproduction of reactive oxygen species (ROS) can cause considerable cellular injuries by attacking nucleic acids, lipids, and proteins. It was argued by Kranner et al. [18] that “effective control of reactive oxygen species and mutual up-regulation of protective mechanisms was critically important for the evolution of lichens, facilitating the transition from free-living fungi and green algae or cyanobacteria to the lichenized state”. On one hand, the role of energy dissipation mechanisms against desiccation stress was investigated by employing chlorophyll fluorescence techniques and the quantification of xanthophylls by HPLC analysis. On the other hand, the response of ROS scavengers-enzymatic and low-molecular weight antioxidants during desiccation/rehydration (D/R) was studied by HPLC, electrophoretical, immunological and spectrophotometrical techniques. Comparative approaches with lichen species differing in DT suggested that, besides the antioxidant system, other mechanisms allowed lichens to survive desiccation [19,20].

The latest reviews on lichen DT (e.g., [10,18,21]) revealed that the knowledge about DT mechanisms was much scarcer than in other organisms tolerant to desiccation such as bryophytes or vascular resurrection plants. During the last decade, this gap has been reduced due to advances in molecular biology, particularly in the use of -omic technologies. The increasing availability of genomic, proteomic, transcriptomic and metabolomic data from lichens and their isolated symbionts have expanded our horizons and helped to gain insight into alternative lichen desiccation-tolerance mechanisms. The aim of this review is to present an overview of our current knowledge about DT in lichens with a particular emphasis on the most recent progress.

## 2. Photosynthesis under Desiccation Stress

Terrestrial green algae, including lichen phycobionts, show a decrease of photosynthesis during desiccation that is rapidly recovered after rewetting. Lichen growth depends on the photobionts photosynthesis rate and this is dependent on the lichen water status [22]. In the dehydrated state photosynthesis is blocked, but pigments can continue absorbing light energy, which may result in photoinhibition and photodamage [23]. On the other hand, when the thallus is fully saturated with water (i.e., the lichen contains more water than necessary to saturate photosynthesis) diffusion of CO_2_ to the photobiont is hindered and consequently, there is a depression of net photosynthesis [24]. Therefore, optimal net photosynthesis in lichens is limited to a narrow range of water content (about 65–90% of maximum water content) [25,26]. Out of this range, light intensities often exceed those that saturate photosynthesis, facilitating ROS formation.

The main physiological mechanism to avoid photooxidative damage is non-photochemical quenching of the energy absorbed by PSII (NPQ). The main component of NPQ in higher plants is the energy-dependent quenching (q_E_). It is assumed that q_E_ affords a protection system for the photosynthetic machinery through the light-dependent formation of Δμ_H+_, activating the xanthophyll cycle [27,28] and/or the action of the PSII-associated protein psbS [29]. The low luminal pH activates violaxanthin de-epoxidase, which converts violaxanthin to antheraxanthin and then to zeaxanthin. The increase of zeaxanthin together with activation of the PSII psbS protein produces an increase of energy dissipation as heat [30,31]. However, in the free-living chlorophyte *Chlamydomonas reinhardtii*, the xanthophyll cycle is not required for survivance under excess light and violaxanthin de-epoxidase activity is not required for the generation all Δμ_H+_ dependent NPQ [32]. Similar results, i.e., existence of the xanthophyll cycle, but a contribution to NPQ lower than in higher plants, were described for other free-living chlorophytes by Masojídek et al. [33]. Besides violaxanthin de-epoxidase, two other proteins are activated by the low luminal pH that generates NPQ: the pigment-binding LhcSR proteins that belong to the Lhc family [34] and the psbS protein that is protonated on two luminal Glu residues and is the main sensor of low pH in vascular plants [35]. The involvement of LhcSR proteins in the generation of NPQ was discovered in *Chlamydomonas reinhardtii* [34], where they play a critical role. LhcSR are homologous to the Lhc proteins and has been suggested that one of its members (LhcSR3) is the energy quencher in *Chlamydomonas* [36]. *lhcSR* genes are widespread in many photosynthetic organisms, including green algae, brown algae, diatoms, nonvascular plants, and even some vascular plants [37]. The psbS protein also belongs to the Lhc family, but it does not bind pigments [36] and has been detected in vascular and non-vascular plants, but its presence in microalgae has been elusive for a long time, until recent results obtained by [38] have demonstrated its transit accumulation and functionality in *Chlamydomonas* under high light. More recent results reported by [39] show that in *Chlamydomonas* under a night-day cycle LHCSRs and psbS are present and NPQ is active consistently throughout the day together with state transitions.

In lichen chlorobionts, the xanthophyll cycle activation does not seem to be a general response to drought conditions. For instance, the lichen *Pseudoevernia furfuracea* and the isolated lichen chlorobionts *Trebouxia excentrica* and *Astherocloris erici* do not increase the xanthophyll deepoxidation state in response to dehydration or rehydration [20,34,40]. Otherwise, in some chlorolichens has been described that the xanthophyll cycle is activated under conditions of dehydration and/or high irradiance [41,42,43]. 

In addition to q_E,_ a second NPQ component has traditionally been attributed to state-transitions and named q_T_ [44,45,46]. State-transitions (ST) regulate the total intake of light energy through a reversible process, balancing the excitation flux between PSII and PSI by shuttling the light harvesting complex (LHCII) [47]. In classical models [48,49], under low light conditions, LHCII antenna are attached to the PSII (state 1, S1), but when the light intensity increases some of the LHCII subunits become phosphorylated, which causes their detachment from PSII reaction centers and attachment to PSI, which is called the transition to state 2 (S2). The kinase (known as STT7 in algae) that phosphorylates the LHCII is activated when the plastoquinone (PQ) pool is over-reduced [49,50,51]. When the pool of PQ is re-oxidized, the kinase activity declines and a permanently active thylakoid peripheral phosphatase (TAP38/PPH1) dephosphorylates LHCII in higher plants [52,53], upon which it migrates back to PSII (S1). In *Chlamydomonas* two phosphatases (PPH1 and PBCP) are homologous to proteins that antagonize the state transition kinases (STN7 and STN8) in *Arabidopsis thaliana*, The transition from state 2 to state 1 was retarded in mutants for *pph1* and in *pbcp*, but both mutants eventually returned to state 1. However double mutants *pph1*; *pbcp* for these phosphatases were able to block the transition from state 2 to state 1. The role of PBCP in Arabidopsis is different to its homologous in *Chlamydomas*, as lack of PBCP does not affect state transitions and it is only when this phosphatase is strongly overexpressed that it has a minor effect on the rate of state transitions [54]. In 1999, Chakir and Jensen [55] found that NaF (a phosphatase inhibitor) had a strong negative effect on the recovery of photosynthesis activity after dehydration in the lichen *Lobaria pulmonaria*. Fluoride is known to inhibit the dephosphorylation of the LHCII complex, hence, these authors stated that ST are activated in response to desiccation/rehydration to protect the lichens against excess of light and irreversible photoinhibition. Gasulla et al. [40] analyzing the relaxation kinetics of fluorescence in the dark, found that the main component of NPQ is ST (about 60% of total NPQ, in the isolated lichen algae *Asterochloris erici*). Recently, it has been demonstrated that an induction of plastoquinone-reductase activity associated with chlororespiration can trigger phosphorylation of LHCII in lichen chlorobionts [56].

In green algae the size of the mobile LHCII may constitute up to 80% of the total LHCII pool [48], much higher than in vascular plants (20–25%). Consequently, it has been proposed that in green algae the major regulators of the photosynthetic machinery are ST instead of q_E_ [31,57]. In algae, the state transitions are associated with a switch between linear and cyclic electron flow [58,59]. The physiological role of ST is, on one hand, to avoid the photoinhibition of PSII, and, on the other, to increase the photochemical rate of PSI to allow an efficient recovery from over-reducing conditions under hypoxia/anoxia, allowing rapid restoration of linear and cyclic electron flows, a critical issue for algal survival in its natural habitat [60,61].

Alternative mechanisms of energy dissipation have been described in mosses and lichens, in which new quenching centers appear to be functional during desiccation [62,63,64,65]. According to Bilger et al. [66], desiccation in green algal symbionts induces a functional interruption of excitation energy transfer between the light harvesting chlorophyll *a/b* pigment/protein complex and photosystem II (PSII), implying a light-independent mechanism as a direct effector. In bryophytes and lichens, an alternative quencher of chlorophyll fluorescence, characterized by a long-wavelength (720 nm) emission, was detected. Veerman et al. [62], employing steady-state, low-temperature, and time-resolved chlorophyll fluorescence spectroscopy, provided evidence of a pigment molecule energetically coupled to PSII having an emission band at 740 nm that dominates fluorescence decay in the lichen *Parmelia sulcata* under desiccation. The presence of this quencher has been also detected [67] by sub-picosecond fluorescence spectroscopy in the dried state of the lichen *Physciella melanchla*, which contains the phycobiont *Trebouxia* sp. Komura et al. [67] proposed the operation of a new type of strong quenching in dried lichens, probably associated with the antenna of PSII. Slavov et al. [68] after an ultrafast fluorescence spectroscopy study in *Parmelia sulcata* and kinetic target analysis suggested that two mechanisms are implied in photoprotection during desiccation. The first proposed mechanism is a direct quenching of PSII by the formation of a fluorescent (maximal emission at 740 nm) chlorophyll-chlorophyll charge-transfer state. The second mechanism involves a spillover (energy transfer) from PSII to PSI facilitated by a rearrangement of thylakoid membranes during desiccation, which would bring PSII and PSI units into direct contact.

Heber et al. [69] offered additional evidence for alternative mechanisms of energy dissipation, identifying in dehydrated mosses and lichens a fluorescence quenching independent of light activation. Heber and co-authors [64,69] subsequently accumulated convincing evidence for the existence of alternative pathways for the dissipation of light energy. In lichens subjected to drying, chlorophyll fluorescence decreases and the stable light-dependent charge separation in the reaction centers (RCs) of the photosynthetic apparatus is lost. The removal of structural water is thought to produce a conformational change, such that energy is redirected to alternative sinks where the absorbed light energy is very rapidly (within a picosecond or a few femtoseconds) converted into heat, thus depriving functional RCs of energy and protecting them against photoinactivation [64,67].

Less is known about the protection mechanisms of the photosynthetic machinery of lichens under hydrated conditions. Usually the duration of the hydrated state is short, and the water content of the thallus is constantly changing, as it equilibrates with the water potential of the surrounding atmosphere. Lichen growth depends on the photobionts photosynthetic rate and this is dependent on the lichen water status [22]. The recovery of photosynthesis after desiccation also needs a period that can take from minutes to hours [20]. Guéra et al. [65] proposed a new alternative mechanism to prevent photooxidative damage in hydrated lichen chlorobionts by the transformation of closed reaction centers into energy sink RCs (sRCs) capable of dissipating excess light energy as heat. Closed RCs are the same that reduced RCs, i.e., centers where Qa is reduced and the electron transport from P_680_ is blocked. The conversion from closed RCs to sRC requires a structural, biochemical or conformational change, and once converted, they act as efficient excitation traps that dissipate the energy as heat to avoid damage to the photosynthetic machinery. This model could be related to ST since in green algae LHCII can remain detached from both photosystems, being self-aggregated in a separate LHCII pool where LHCII fluorescence (excited state) is quenched [70]. Aggregation of monomeric and trimeric LHCII subunits creates efficient excitation traps that not only quench excitations in the complexes in which they are located, due to excitation energy transfer between monomers and trimers, but also quench excitations in connected LHCIIs [71].

## 3. Antioxidant Protection

A consequence of desiccation is the increase in the formation of ROS (reactive oxygen species) and, therefore, oxidative stress (reviewed by Kranner et al. [18] and Gasulla et al. [21]). These reactive oxygen species are over-produced during drying, especially in the presence of light. On the one hand, free radical production seems to be closely linked to respiration in a process involving desiccation-induced impairment of the mitochondrial electron chain [72]. On the other hand, although carbon fixation is inhibited during desiccation, electron flow through the photosystems continues if the RCS are not closed by an excess of radiation. Excitation energy can be also transferred from photo-excited chlorophyll pigments to ^3^O_2_, forming singlet oxygen (^1^O_2_), while superoxide and hydrogen peroxide can be produced at photosystem II and photosystem I by the Mehler reaction [73,74]. Likewise, rehydration of lichens produces a burst of ROS during the first minutes and then decreases [75,76].

Scavenging of ROS can be carried out by enzymatic or non-enzymatic antioxidants. Antioxidant enzymes include superoxide dismutase (SOD), catalases (CAT), peroxidases (POX) and auxiliary enzymes such as mono- and dehydro-ascorbate reductases and glutathione reductase (GR). From the few studies carried out with lichens, a clear relationship between desiccation tolerance and antioxidant levels have not been found. For instance, Mayaba and Beckett [77] observed that activities of SOD, CAT, and ascorbate peroxidase (AP) were similar during wetting and drying cycles in *Peltigera polydactila*, *Ramalina celastri* and *Teloschistes capensis*, which grow in moist, xeric and extremely xeric habitats, respectively. Kranner [19] neither found correlation between GR activity and the different degrees of desiccation-tolerance of three lichens, *Lobaria pulmonaria*, *Peltigera polydactila* and *Pseudevernia furfuracea*. Weissman et al. [76] reported that *Ramalina lacera* loses almost all CAT activity after rehydration and SOD decreases by 50–70%. Kranner et al. [18] concluded that enzymatic antioxidants were perhaps more likely to be involved in removing ROS produced during normal metabolism or by other stresses rather than during rehydration following severe desiccation.

In contrast, less studied in algae and lichen than in plants or animals, but potentially important, is nitric oxide (NO). The first work that focused on NO production in lichens was published by Weissman et al. [76], who carried out a microscopy study of *Ramalina lacera*. These authors described the occurrence of intracellular oxidative stress during rehydration together with the release of NO by the mycobiont, but not by the photobiont. Catalá et al. [78] described that mycobiont derived NO had an important role in the regulation of oxidative stress in *Ramalina farinacea* and in the photooxidative protection of photobionts in lichen thalli, mainly in the early stages of lichen rehydration. More recently Expósito et al. [79] have shown the existence of nitrate reductase (NR) activity correlated with NO generation in the lichen *R. farinacea* under stress conditions. NR activity seems to have an important role in the cortex hyphae and in phycobionts in the first hours after rehydration.

The responses to desiccation stress in whole lichens are different from those happening in their isolated photobionts [80]. Desiccation/rehydration cycles cause a massive accumulation of ROS, especially during the beginning of rapid drying and rehydration in lichen microalgae [76,78]. Differential desiccation tolerance across green algal species, assessed by photosynthetic efficiency during D/R cycles, was accompanied by differential accumulation of intracellular ROS [81] and involves specific priming of the antioxidant system, which seems to be related to habitat preferences [82] and symbiotic habit [83]. Gasulla et al. [40] reported that SOD and POX activities decreased during desiccation and early recovery in *Asterochloris erici*, while dehydrins were constitutively expressed. *Coccomyxa simplex* (Csol) produces ascorbate and increases its synthesis upon D/R, while it is absent in *Trebouxia* sp. (TR9) isolated from *Ramalina farinacea* [84]. *Ramalina farinacea* is a Mediterranean fruticose epiphytic lichen adapted to xeric habitats, while Csol is the phycobiont of *Solorina saccata*, a foliaceous lichen that grows in humid rock crevices. It has been reported that ascorbate, a water-soluble low-molecular weight antioxidant, plays an important role under conditions that favour oxidative stress, such as excessive light or atmospheric pollution [85,86]. TR9 shows higher amounts of simple phenolics and soluble carbon compounds, mainly polyols, than Csol. Simple phenolics and polyols constitute important groups of plant secondary metabolites with antioxidant properties [87]. Thus, ascorbate would play a main role in the antioxidant protection of Csol whereas polyols and phenolics could act as ROS scavengers, avoiding oxidative damage in TR9. Accordingly, recent results with the resurrection plant *Haberlea rhodopensis* demonstrated that the polyphenolic antioxidant and antioxidant enzymes involved in the ascorbate-glutathione cycle increased during desiccation [88]. Similarly to results of Centeno et al. [84] and Bertuzzi et al. [89] comparing the responses of two lichens with different ecology (*Parmotrema perlatum*, hygrophilous, and *Xanthoria parietina*, meso-xerophylous) exposed to oxidative stress induced by ozone and to wet or dry conditions, showed that both species differed in their antioxidant profile (ascorbate was higher in *X. parietina*, glutathione in *P. perlatum*), and in the activity of ROS-scavenging enzymes, more intense in the hygrophilous *P. perlatum* than in the meso-xerophilous *X. parietina*. These authors concluded that lichens are ozone-tolerant thanks to the constitutive antioxidant systems, intimately related to their poikilohydric lifestyle, because most of the examined parameters were more heavily affected by water availability and particularly by air humidity than by ozone exposure.

Following comparative studies of Csol vs. TR9 subjected to several D/R cycles, Hell et al. [82] showed that manganese superoxide dismutases (MnSODs) were induced in both algae after desiccation and decreased after rehydration. However, CAT2 and GR activities only increased in TR9. Here, these activities were maintained after four cycles of desiccation/rehydration, but ascorbate peroxidase activity was detected only in Csol. Transcript levels of antioxidant enzymes indicate that most isoforms of MnSOD and FeSOD were induced by desiccation and repressed after rehydration. *CAT2* gene expression was also upregulated and maintained at higher levels even after four cycles of desiccation/rehydration in accordance with enzymatic activities. The authors propose that the lack of antioxidant response to desiccation previously reported in isolated lichen algae may have occurred because algal cultures were only subjected to a single desiccation/rehydration cycle, which probably was not enough to acclimate them to these changing conditions.

## 4. The Cell Wall and Extracellular Polymers in the Tolerance to Desiccation

One of the most remarkable results of dehydration is the progressive reduction of the protoplast volume [90], which can disrupt the subtle and complex connections between the plasmalemma and the cell wall (CW). During evolution, resurrection plants and some charophyte algae have developed flexible cell walls which can couple their own contraction to the protoplast shrinkage during drying and to re-expand after rehydration [90,91]. A well-documented case is *Craterostigma wilmsii*, a resurrection plant in which the flexibility of their cell walls seems to increase under drying conditions due to changes in the glycosidic composition and molecular size of xyloglycans [92]. Another well-known case is the green algae *Klebsormidium* in which the cell walls undergo a controlled collapse and changes in thickness during drying [23]. In chlorolichens, ultrastructural studies performed by Honegger et al. [93], employing scanning electron microscopy (SEM) and transmission electron microscopy (TEM) after cryofixation, showed notable prototoplast contraction during dehydration in both mycobiont and photobiont cells. However, protoplasts maintained close contact with the cell walls indicating a drying-driven deformation of the cell walls. In the last few years, González-Hourcade et al. [94] have searched for possible D/R-induced structural alterations in two phycobionts of lichens that grow in contrasting habitats (TR9-mediterranean- and Csol-relatively wetter environments-), especially in their CW and plasma membrane context through different microscopy approaches: TEM, low-temperature SEM and environmental SEM (ESEM). All ultrastructural analyses consistently showed that desiccation caused progressive cell shrinkage and deformation in both microalgae, which could be rapidly reversed when water availability increased. However, ESEM experiments suggested that Csol does not tolerate drastic desiccation conditions, requiring fairly high relative humidity (RH >55%) during desiccation to recover upon rehydration, while TR9 is better adapted to rapid changes in water status. Irrespective, when desiccation occurs at a RH/rate such as that encountered in their respective habitats, the CW of both TR9 and Csol deforms accompanying the protoplast shrinkage/expansion during D/R.

Early studies on drying seeds by Webb and Arnott [95] revealed that the “controlled collapse” of the CW was necessary to maintain structural organization and cell viability in the desiccated state. Importantly, they found that CW deformation occurred in a species-specific manner and was related to the biochemical composition of the CW. This notion has now been extended to other desiccation-tolerant organisms [96]. Few studies have been conducted on CW polysaccharide composition in lichen-forming algae. Besides a very low content (or even absence) of cellulose, the predominance of β-galactofuranans has been reported in the photobionts of *Ramalina gracilis* and *Cladina confusa* [97,98]. More recently, the ultrastructure and polysaccharide composition of the CWs of *Trebouxia jamesii* and TR9, the phycobionts of *R. farinacea*, were analysed [99]. At the ultrastructural level, four clearly differentiable layers in the *T. jamesii* CW were observed, whereas TR9 showed a more diffuse structure in which only three layers could be distinguished. Fractionation of *T. jamesii* and TR9 CWs revealed a high proportion of galactose, xylose and rhamnose that was associated with a β-xylorhamnogalactofuranan present in the hot water fraction. Meanwhile, the alkaline fraction showed high proportions of galactose, glucose and mannose. In addition, a comparative analysis between TR9 and, in this case, Csol CWs indicated that although both CWs had the same number of layers (three), these were thicker and more diffuse in TR9 and thinner and more defined in Csol than in TR9 [94,99,100]. Furthermore, glucose, mannose, galactose and rhamnose were the predominant monosaccharides in Csol, in contrast to the glycosyl composition of the TR9 CW. In the last few years, González-Hourcade et al. [101] have studied in detail the possible changes in the main polysaccharides of TR9 and Csol CWs after exposure to D/R. Notably, cyclic D/R had a high impact, since it strongly altered the size distribution of TR9 hot water-soluble polysaccharides mainly composed of a β-3-linked rhamnogalactofuranan and Csol KOH-soluble β-glucans. It is suggested that such biochemical remodeling of the CW induced by D/R could increase CW flexibility (acclimation effect), facilitating regulated shrinkage and expansion of D/R-tolerant microalgae.

Another important aspect of DT in lichen microalgae is related to the extracellular polymers (EPS), excreted by the cell and retained on the outer face of the cell wall [102]. Extracellular polysaccharides usually account for at least 50–60% of total EPS. They are long-chained and composed of repeating units of neutral monosaccharides, usually glucose, galactose, arabinose, mannose or fucose [103,104]. The amount and composition of EPS depends on the microalgal species and seems to be also influenced by their physiological performance, since abiotic stresses [105] such as desiccation or nitrogen starvation, but also non-stressful treatments such as exposure to red and blue light [103] increase the production of EPS. Microalgal EPS also contain low proportions of uronic acids, and organic (methyl, amino) and inorganic (sulphate) functional groups as chemical substitutions in sugar units of polysaccharides that can affect their physical and biological properties. Sulphated polysaccharides (SPs), which contain some of their sugar residues as half esters of sulphuric acid, are widely distributed in EPS of a variety of organisms including mammalian [104]. SPs (along with other charged groups) can hold water molecules, thus increasing the hydrophilicity of EPS and contributing to their gel-like appearance. More importantly, this higher water retention capacity could be crucial to avoid sudden changes in the cell hydric status in organisms subjected to cyclic D/R conditions such as lichen microalgae. However, the characterization of EPS in aeroterrestrial microalgae and their possible biochemical changes under abiotic stress is only scarcely known. Recent studies [84,101,106] analyzed the glycosyl composition and linkage of the extracellular polysaccharides in fresh cultures of TR9 and Csol. TR9 polysaccharides were mainly composed of 50% galactose and 28% glucose with lower proportions of rhamnose, mannose, arabinose, fucose and xylose. Csol also contained polysaccharides mainly composed of galactose (ca. 50%). However, in Csol EPS mannose, glucose, and rhamnose are similarly abundant and in a proportion significantly higher (13–15%) than in TR9 EPS, except in the case of glucose that considerably decreased. In summary, TR9 EPS conained galactans as the predominant polysaccharides [106]. In contrast, Csol EPS were much more heterogeneous, constituted by a complex mixture of polysaccharides. The exposure to species-specific conditions of D/R (dehydration at 25% RH or at 55% RH for TR9 or Csol, respectively) induced quantitative and qualitative changes in the EPS polysaccharides of TR9 and Csol. Their polydispersed molecular mass distribution was not altered by D/R in either microalgae, but the abundance of several polysaccharides was notably changed [101]. However, in Csol, the medium-low-sized uronic acid-containing polysaccharides present in hydrated cells were substituted by higher molecular mass carbohydrates after D/R. Both microalgae contained sulphated polysaccharide(s); however, they increased after cyclic D/R only in TR9.

The CW of vascular plant cells also includes structural and water-soluble proteins (WSP). They are mainly involved in CW structure, modifications of CW components, signalng and interactions with plasma membrane proteins at the cell surface. Three major classes of structural CW proteins have been recognized to date: extensins, proline-rich proteins (PRPs), and glycine-rich proteins (GRPs) [107]. In addition, there is increasing evidence demonstrating that genes coding enzymes involved in the synthesis or remodeling of the CW components of vascular plants are differentially regulated by diverse stresses, suggesting that they may facilitate stress tolerance through changes in the biochemical and mechanical properties of the CW (reviewed by Houston et al. [108]). It is highly probable that the CW of lichen microalgae also contain structural and WSP, and that they play important roles in CW structure and remodeling. At present, only two pieces of evidence of CW-associated proteins in lichen microalgae have been published. The first one was obtained by König and Peveling [109] using cytochemical methods and electron microscopy that showed the presence of structural proteins in the CW of several photobionts. Recently, González-Hourcade et al. [110] characterized by LC-MS/MS the EPS-associated proteome of TR9 and Csol phycobionts under control conditions and after cyclic D/R. Both exoproteomes consisted of a similar number of extracellular proteins (111 in TR9 and 121 in Csol) and shared a variety of putative biological functions (hydrolases, ROS scavenging, cell-cell interaction, etc.) which seem to be oriented to preserve or adapt the extracellular structures to cyclic drying. However, all Csol exoproteins showed a constitutive expression while approximately 20% of exoproteins of TR9 were inducible by D/R, probably reflecting the harsher hydric conditions in which this latter alga thrives.

## 5. Cytoplasm Vitrification and Molecular Stability

A critical process that occurs in the cells of anhydrobiotic organisms during dehydration is the vitirification of the cytoplasm at room temperature. As water content decreases, the cellular fluids become increasingly viscous, which reduces molecular mobility and increases the possibility of molecular interactions. When the cytoplasm dries to <0.3 g H_2_O g dry weight (DW)^−1^ the mobility of the molecules is five orders of magnitude lower compared to the liquid state [111], reaching the so-called “rubbery” state. With a further water loss (0.1 g H_2_O g DW^−1^) the cytoplasm vitrifies and enter into the “glassy” state, an amorphous metastable state that resembles a solid, but maintains the physical properties and disorder of a liquid [112]. The transition to the glassy state reduces drastically the molecular mobility (equivalent to a flow rate of 0.3 μm year^−1^), which nearly stops any chemical reaction including metabolism, but also those that lead to deterioration and ageing [18]. Life span of desiccation-tolerant plants is inversely correlated with intracellular molecular mobility that depends on water content, temperature and cytoplasmic components [113]. Thus, the vitrification of the cytoplasm seems to be a requisite for long-term survival of anhydrobiotic organisms. The glassy state has mainly been studied in seeds and pollen; however, little is known about this process in lichen symbionts. Recently, Carniel et al. [114] have demonstrated that vitrification also occurs in lichens upon desiccation. The authors observed that the molecular mobility in the lobes of the lichen *Flavoparmelia caperata* dried at 5% RH, was lower than in those dried at 75% RH. In addition, while the xanthophyll cycle remained active in thalli exposed to 75% RH, de-epoxidation of violaxantin to zeaxanthin did not occur at 5% RH. According to their results, the transition to the glassy state occurred between 0.12–0.08 g H_2_O g DW^−1^ in the lichen *F. caperata* at 20 °C. Interestingly, lobes stored for 45 days at RH >55% RH (i.e., WC > 0.12 g H_2_O g DW^−1^) suffered damage such as bleaching and incomplete photosynthesis recovery, which indicates that vitrification plays a pivotal role in the survival of lichens during long desiccation periods.

In desiccation-sensitive organisms, the disappearance of the water shell around macromolecules causes the alteration of the hydrophobic and hydrophilic relations that can initiate protein aggregation and membrane fusion. However, in anhydrobiotes the native cellular structures are maintained in the glassy state during desiccation and their functionality is recovered upon rehydration. The stabilization of proteins and other macromolecular structures in desiccation-tolerant plants and seeds is achieved by means of metabolic adjustments including the synthesis of non-reducing sugars, late embryogenesis abundant proteins and heat shock proteins [115].

A general response of resurrection plants to dehydration is the accumulation of sugars. Sucrose is the most common sugar that accumulates during desiccation, although other species-specific diversity of sugars can be also synthetized, e.g., octulose in *Craterostigma plantagineum*, trehalose in *Myrothamnus flabellifolia*, raffinose in *Xerophyta villosa*, galactinol in *Xerophyta viscosa* reviewed in Zhang et al. [116]). The accumulation of carbohydrates facilitates the formation of glasses because they can generate hydrogen bonds. The glass-to-liquid transition temperature, Tg, increases with the molecular weight of the carbohydrate, allowing the formation of glasses at an appropriate temperature and water content. In addition, carbohydrates can establish hydrogen bonding with the polar regions of proteins and phospholipids of the membranes replacing the structural function of water, the so-called water replacement hypothesis [117]. Lichens that establish symbiosis with cyanobacteria accumulate sugars (glucose), but those containing green algae accumulate preferentially polyols (ribitol, arabitol, erythritol, sorbitol, etc.) (Palmqvist et al. [118] and references therein). Sugar alcohols (polyols) play a role as organic osmocompatible solutes, stabilizers of proteins, and serve as rapidly available respiratory substrates for metabolism maintenance under stress and repair during recovery in aeroterrestrial algae (Holzinger and Karsten [23] and cites therein). The carbohydrates produced by the photobiont are transferred to the mycobiont that transform them into mannitol [119]. The polyol content of lichens varies widely depending on the species and season, but can represent from 2% to 36% of the thallus dry weight [120,121,122], levels that are similar to the concentration of sugars in the dried leaves of resurrection plants [123,124,125]. Polyols can act as macromolecular stabilizers since they contain multiple hydroxyl groups that can substitute the hydrogen bonds of water during desiccation. However, in contrast to resurrection plants, the amounts of polyols are similar in wet and dry thalli [120,122,126], which might provide a permanent protection against injures caused by desiccation stress in lichens. There are only a few metabolomic studies on lichen microalgae, which have essentially been limited to primary metabolism and sugar-related metabolites. One of these studies has shown that the DT of *U. lambii’s* photobiont might be related to the constitutive accumulation of ribitol and sucrose [127]. Even more exiguous are the studies on the effects of cyclic desiccation on the metabolic profile in lichen algae. Centeno et al. [84] compared the profile of primary metabolites (mainly sugars and respiratory intermediates), phenolics and sugar-derived metabolites in the ecologically contrasting phycobionts TR9 and Csol exposed to one cycle of D/R. The most striking feature was a decrease in primary metabolism during desiccation in both algae, and a differential metabolic species-specific profile. TR9 showed higher amounts of simple phenolics and soluble carbon compounds than Csol. A detailed analysis of the metabolic profile of simple sugars and polyols in TR9 and Csol along four cycles of D/R highlighted the important role of these metabolites in the protection against cyclic desiccation in both microalgae [128]. Csol showed lower constitutive level of the polyol pool but induced its synthesis under D/R, whereas TR9 constitutively accumulated higher amounts of these metabolites. In Csol, mannitol was the most abundant polyol in fully hydrated cells and its abundance remains approximately constant along D/R cycles. Additionally, the proportion of mannose, maltose and sorbitol (which was not detected in control cells) increased after the second D/R cycle. In TR9, arabitol is the major sugar-alcohol of fully hydrated cells, but its proportion decayed during D/R, as sorbitol increased, suggesting a shift in polyol metabolism induced by cyclic desiccation, accumulating a more potent osmoprotectant without modification of the overall size of the polyol pool [128].

Late embryogenesis abundant proteins (LEAs) are so-called because they are accumulated during the late phases of orthodox-seed development just before drying [129]. They are also accumulated in the leaves in response to dehydration, especially in resurrection plants [130]. For this reason, LEA proteins have been directly related to the acquisition of DT. LEA is a family of cytosolic proteins that are intrinsically unstructured in aqueous solution, but many assume their native conformation during drying, are heat-resistant, and contain a high proportion of hydrophilic amino acids. These molecular characteristics may allow formation of a coat around macromolecules to maintain a cohesive water layer during water loss [131,132]. On further dehydration, the hydroxylated residues of LEAs would substitute the hydrogen bonding of water to stabilize other protein and lipids [133]. In vitro experiments have demonstrated that LEA proteins can prevent the aggregation of proteins due to desiccation, a protective role that is augmented in the presence of non-reductant disaccharides [134]. In addition, the glass matrix formed by the combination of LEA proteins and carbohydrates results in stronger hydrogen bonds and higher Tg values than separately [135]. The knowledge about LEA proteins in lichens is very scarce and, indeed, the few studies have focused only on photobionts. Carniel et al. found two genes belonging to group 3 LEAs and one dehydrin (group 2 LEAs) in the isolated phycobiont *Trebouxia gelatinosa*, whose level of transcription did not change in response to desiccation or the following rehydration. These results agree with the constitutive accumulation of dehydrins in the lichen microalgae *Asterochloris erici* that is not affected by D/R [40]. Cytoplasmic dehydrins have been located in endomembrane sheaths, which suggest that they may be involved in the stabilization of these membranes [136]. In presence of sucrose, LEAs form amphipathic α-helix during drying [137], thus, whereas the hydrophobic face of dehydrins might interact with lipids, the highly polar segments of dehydrins might establish polar hydrogen bonds with other macromolecules or with compatible solutes of the cytosol [136].

The synthesis of oligogalactolipids (OGL) is another mechanism that might collaborate with LEA proteins in the preservation of membranes in the glassy state. Oligogalactolipids are glycolipids with a head-group formed by three to five galactoses. They are accumulated in the chloroplast of vascular plants, both desiccation sensitive or desiccation tolerant ones, in response to different kinds of osmotic stresses such as freezing [138,139,140], drought [141], desiccation [142] or salinity [141], while they are constitutively present in desiccation tolerant poikylohydric plants such as mosses and algae [143]. Several protective roles have been proposed for OGL: (1) the large polar head of OGL may increase the repulsive force between adjacent thylakoidal membranes to avoid their fusion during cellular collapse [138,142]; (2) the hydroxyl groups of the galactoses could specifically interact with LEA proteins [112] contributing to membrane stabilization [144]. In addition, desiccation triggers the degradation of monogalactosyldiacilglycerol (MGDG) in the lichen phycobionts [145]. This is a universal response of plants to any kind of osmotic stress [143] because MGDG is a cone-shaped lipid that forms inverted hexagonal structures and destabilizes the membranes during dehydration [138].

Heat shock proteins (HSP) are also involved in the conservation of the structures and functionality of cellular macromolecules during desiccation. As their name indicates, they were first observed in response to high temperatures, but they are also induced after the exposure to other kinds of abiotic stresses, such as dehydration. HSPs are a universal superfamily of conserved proteins that can be broadly divided in ATP-dependent large HSP (lHSP) and ATP-independent small HSP (sHSP), classification that is not only based on their molecular weight but also on their mode of action. The large heat-shock proteins-such as the HSP90 and HSP70- are also known as chaperonins, they help in the correct folding of newly synthesized proteins and the refolding of denatured proteins, being also involved in processes of protein transport [146]. On the other hand, sHSP contain α-helix that can bind the misfolded proteins by selective interactions with their hydrophobic surface preventing their further aggregation [147,148,149]. In addition, in plants and in yeast, sHSP are associated with membranes in fresh and in desiccated state, where they may stabilize the lipids during cytoplasm vitrification [150,151]. In the lichen phycobionts *Asterochlori erici* up-expression of the *Hsp90* gene was observed during dehydration, resulting in the accumulation of the respective protein [152]. The large HSP 70 is constitutively present in *Trebouxia gelatinosa* and does not change its amount in response to desiccation and rehydration [153]. However, its transcripts level decreases during dehydration [153,154]. Regarding sHSP, it is only known that the transcription of the HSP20 is inhibited during drying [154]. Further studies are needed to determine the exact role of chaperones in the adaption of lichens to desiccation.

## 6. Additional Mechanisms of Desiccation Tolerance Revealed by Transcriptomic Approach

High-throughput sequencing technologies offer significant advantages in elucidating biological processes underlying specific physiological responses of organisms. These include RNA-Seq technology, which provides a high coverage and resolution in transcriptomic approaches allowing the quantification of gene expression levels. The scarcity of available genomic and/or transcriptomic information for non-model photosynthetic organisms makes it extremely difficult to study the molecular mechanisms underlying DT in green algae and lichen phycobionts in particular from a holistic perspective. However, in addition to the changes in the transcriptional expression of genes involved in the DT of lichen algae outlined in the preceding sections, recent global transcriptomic approaches have revealed the differential expression of hundreds of genes in lichen-forming chlorophyta algae undergoing desiccation and/or rehydration treatments (e.g., *Trebouxia gelatinosa* [154], *Asterochloris glomerata* [155] and the chlorobionts of *Cladonia rangiferina* [156] and *Cladonia grayi* [155]). Studies on free-living chlorophytes demonstrate that both desiccation tolerant and sensitive species share up-regulation of protective genes but only desiccation tolerant algae show down-regulation of certain genes related to various metabolic processes during the desiccation time course [81]. The authors suggest that the extensive down-regulation of gene expression in desiccation tolerant algae results in a metabolic slowdown affecting the generation of precursor metabolites and energetic metabolism. Regarding photosynthetic activity of chlorophyta algae in response to dehydration, two different scenarios have been shown depending on their tolerance to desiccation [81]. One of them consisting of a generalized down-regulation of genes related to photosynthesis in response to desiccation stress observed in the desiccation tolerant free-living chlorophytes *Acutodesmus deserticola* and *Flechtneria rotunda*; and the other one consisting of the up-regulation of the same genes observed in the closely-related aquatic algae *Enallax costatus*. However, in the desiccation tolerant lichen-forming chlorophyte *Trebouxia gelatinosa*, genes related to photosynthesis are strongly over-expressed in response to desiccation indicating a massive up- regulation of the photosynthetic machinery in this algal species [154]. Similarly, in *Asterochloris* sp., which is another desiccation tolerant lichen-forming chlorophyte, several genes coding for products involved in photosynthesis are up-regulated in response to desiccation [156]. The presence of abundant transcripts coding for components of the photosynthetic apparatus upon rehydration in *Trebouxia gelatinosa* has been interpreted as a mechanism contributing to the fast re-establishment of photosynthesis [154]. The fast re-establishment of the photosynthetic activity is expected to require a highly efficient NPQ, which is the main physiological mechanism to avoid photooxidative damage. This assumption is supported by the demonstration that desiccation-induced quenching occurs in desiccation tolerant species of aero-terrestrial species including isolated lichen algae, but not in desiccation sensitive species [157]. In general, desiccation-tolerant streptophyte algae [158,159] and angiospems [160] increase the transcript pool of ROS scavenging proteins in response to desiccation stress. However, a different picture is observed in the lichen chlorobiont *Trebouxia gelatinosa*, in which the expression of most of the primary ROS scavenging enzymes remains steady upon desiccation [154]. A similar scenario can be deduced from the analysis of gene expression data in *Asterochloris* sp., which is the chlorobiont of the lichen *Cladonia rangiferina* [156]. As indicated earlier, the flexibility of the cell wall due to desiccation-induced ultrastructural changes and biochemical remodeling of polysaccharides has been proposed as a major contribution to DT in both free-living and lichen-forming chlorophytes [110,161]. In the lichen alga *Trebouxia gelatinosa*, the genes coding for expansins are up-regulated upon dehydration. Carniel et al. [154] proposed that it is a possible mechanism directing to the remodelling of the cell wall. Differential gene expression analyses of *Cladonia grayi* symbionts in response to desiccation indicate that both the fungus (*Cladonia grayi*) and the alga (*Asterochloris glomerata*) up-regulate genes coding for proteins involved in cell wall turnover and extracellular hydrolases [155]. The ability of some organisms to recover from desiccation is generally dependent on a slow dehydration rate because fast dehydration affects membrane integrity causing intracellular solute leakage after rehydration [162]. Thus, changes in membrane permeability to water can contribute to avoid damage to the membranes during the D/R time course. In the lichen chlorobiont *Trebouxia gelatinosa*, aquaporins are significantly up-regulated in dehydrated samples [154]. In the phycobiont of the lichen *Cladonia rangiferina* transcripts coding for a protein similar to an aquaporin with glycerol transport activity in *Chlamydomonas reinhardtii* (GenBank accession XP_001694120) are more abundant upon desiccation [156]. In the charophyte *Zygnema circumcarinatum* two transcripts coding for aquaporins are highly up- regulated upon desiccation [159]. These results, taken together, suggest an active role of aquaporins in DT of green algae. Desiccation related proteins (DRPs) have also been suggested to be involved in DT despite their function remaining mainly hypothetical. DRPs were first described in the resurrection plant *Craterostigma plantagineum* [163], the presence of *DRP* genes has been also reported in non-resurrection plants e.g., *Gossypium barbadense* [164] and *Oryza sativa* [165] and *Arabidopsis thaliana* [166]. Quantitative analysis of transcripts for the *DRP* gene pcC13-62 of *Craterostigma plantagineum* in two closely related species with contrasting DT, indicates that their levels are much lower in the desiccation-sensitive than in the desiccation-tolerant species confirming its role in DT [167]. DRPs have also been found in lichen chlorobionts from three different genera (*Asterochloris*, *Coccomyxa* and *Trebouxia*). Genes coding for DRPs in chlorophytes and embryophytes are not orthologous and some of them probably have been horizontally transferred from bacteria involved in the lichen symbiosis [155]. Moreover, DRPs are highly diversified in the lichen alga *Trebouxia gelatinosa*, in which most of DRP transcripts increase during dehydration and decrease upon rehydration, which highlights the importance of DRPs in the response of this algal species to desiccation and rehydration [154]. In light of these findings, it is critically important to increase the production of molecular data from a greater number and diversity of aero-terrestrial green algae, including both desiccation-tolerant and desiccation-sensitive species/strains.

## 7. Regulation of Cellular Responses

Little is known about how the lichen symbionts detect water loss and translate this signal into a physiological response. The abscisic acid (ABA) signal transduction pathway is directly related with the acquisition of DT in seeds [168] and in the vegetative tissues of resurrection plants [169]. This phytohormone is a central regulator of the response to various forms of environmental stresses, such as cold, osmotic and saline stress [170,171]. Presence of ABA is widespread in microalgae [172] including both free-living and lichen-forming chlorophytes [173,174] and common in lichens: it has been detected in more than 50 lichen species (see Hartung [175] for a review). ABA signal transduction occurs through the PYR/PYL/RCAR components of the ABA receptors; 2C protein phosphatases as negative regulators; and SNF1-related protein kinases 2 (SnRKs) that are positive regulators. In addition, ABA signaling pathway is interconnected with mitogen activated protein kinase (MAPK) signaling cascades in plant species [176]. In the lichen chlorobiont *Trebouxia* sp. TR9, the ABA transduction pathway is probably functional since ABA-related genes-including some MAPKs are up-regulated in response to exogenous ABA [173]. In plants, the accumulation of ABA triggers a major change in the transcription of genes and the induction of physiological responses, such as stomatal closure [177] or the promotion of seed DT and dormancy [178]. Some effects of ABA on chlorophytes include the activation of antioxidant tolerance responses by increasing antioxidant enzyme activity [179] and stimulating the synthesis and accumulation of carotenoids in the chlorophyte *Dunaliella salina* [180]. ABA seems to be involved in the response to desiccation stress in intertidal seaweed (Rhodophyta) species [181] and to salt stress in the chlorophyte *Dunaliella salina*. Nonetheless, the role of ABA is not clear in lichens since it accumulates transiently during rehydration instead of during dehydration [182], as happens in vascular plants and bryophytes. In addition, it has been reported that the exposure of *Trebouxia* sp. TR9 to high NaCl concentration (up to 5 M) does not cause significant changes in either the phytohormone cell content or the expression of ABA-related genes [173]. These results indicate that lichen phycobionts may employ alternative molecular pathways to cope with osmotic stress.

The phospholipase D (PLD) signaling pathway has been proposed to be involved in the post-translational control of rapid osmotic stress-induced phycobionts responses through the activation of MAPK-like cascades [145]. The formation of phosphatidic acid (PA) as a result of the activation of PLD is among the primary events (within minutes) upon the detection of cell water loss [183]. PA acts as second messenger in the modulation of plant functions by recruiting target proteins to particular membranes and/or influencing their activity, e.g., acting as an activator of MAPKs [184]. Gasulla et al. [145] found several pieces of evidence that indicated the involvement of PLD and MAPK pathways in the control of cellular responses to hyperosmotic stress in the lichen algae *Asterochloris erici*: (1) PA was synthesized during desiccation; (2) PLD inhibition reduced the capacity to recover the photosynthesis activity upon hyperosmotic stress; (3) PLD was related with the activation of MAPK-like proteins; (4) Several proteins were phosphorylated during desiccation. Enzymatic addition of phosphate groups by kinases modulates protein function and, therefore, physiological responses. Thus, the activation of the PLD-pathway during the early dehydration stages and the subsequent activation/inhibition of enzymes by phosphorylation might help lichens face the frequent and rapid changes of water content that they undergo in their natural habitats.

The gas nitric oxide (NO), besides its possible antioxidant role, might also play an important function in the regulation of the defensive response of lichens against desiccation stress. NO is a key intra- and intercellular signaling molecule in a very broad range of organisms, from animals to plants [185]. In the latter, NO has been found to be involved in the control of diverse biochemical and physiological responses to stress such as pathogens [186], salt [187], temperature [188] and drought [189]. Downstream, NO can lead to post-translational modification of proteins through S-nitrosation [190] and tyrosine nitration [191], tuning their enzymatic activities. The biosynthesis of NO in plants can be carried out by two different enzymes, nitric oxide synthase (NOS) [192] and nitrate reductase (NR) [193]. Expósito et al. [79] observed that the specific inhibition of NR suppressed NO synthesis in the lichen *Ramalina farinacea* during dehydration, whereas the use of a NOS inhibitor did not reduce the production of NO. The activity of NR in *R. farinacea* was assessed as 91 µU/mg protein, a level comparable to other organisms. In lichens, rehydration causes a peak in the production of NO, which is mostly produced by the mycobiont [76,78,194], although, in a minor proportion, phycobionts are also able to synthesize it [128]. It has been proposed that, as a gas, mycobiont derived NO can diffuse across the lichen thallus and induce the antioxidant response of the algal partner [78,79]. Recently, Hell et al. [128] have reported that in lichen phycobionts there is a close relationship between the levels of NO and the transcription of genes involved in the synthesis of polyols. These results reinforce the hypothesis that NO may act as a signaling molecule, facilitating osmoregulation and antioxidant protection in lichens. However, further studies are required to distinguish between the antioxidant and the signal roles of NO in the response of lichens to desiccation and rehydration.

Independently of which signal pathways controls the lichen response, desiccation triggers changes in the expression pattern for hundreds of genes in the isolated lichen-alga *Trebouxia gelatinosa* [154], as well as in the whole lichen *Cladonia rangiferina* [156]. However, there is no evidence that the deep modification of the transcriptome causes a proportional change in the proteome. In the lichen phycobiont *Asterochloris erici* only 11 proteins were up-regulated and 54 down-regulated-among the 500 spots detected in the proteomic analysis- in response to dehydration [152]. Moreover, a correlation between transcription and gene expression has been only demonstrated in a few cases, such as the synthesis of the HSP90 [152], the induction of some antioxidant enzymes [82], or some polyol metabolism enzymes [128]. Thus, the induction of protective mechanisms in response to desiccation could not be attributed only to de novo synthesis of proteins. The accumulation of transcripts during dehydration has been postulated to be the result of an increase of stability of selected mRNA instead of de novo synthesis [152]. This hypothesis is based on studies carried out in another poikilohydric organism, the moss *Tortura ruralis*, in which, desiccation triggers changes in the abundance of numerous transcripts, but protein synthesis declines almost immediately during dehydration [195]. In this moss, some transcripts are stabilized by sequestration in mRNP particles in the dried gametophytes [196]. Presumably, the selected mRNAs are then used during rehydration to activate repair mechanisms or to prepare the cell for a subsequent desiccation. Regulation of gene expression at the translational level would be obviously more rapid than a response at the transcriptional level. This strategy may explain the priming of some protective responses recently described in the lichen phycobionts Csol and TR9 [82,128], since the progressive accumulation of selected mRNA during consecutive cycles of D/R may allow faster and more intense responses.

## 8. Concluding Remarks and Future Perspectives

During the last decade there has been a quantitative and qualitative leap in the knowledge of DT in lichens. This advance has been possible because of the onset of the use of “omics” techniques including genomics, proteomics, metabolomics and transcriptomics. These techniques have allowed us to deepen our knowledge of the role of the antioxidant system, sugar and polyol metabolism, and photoprotection mechanisms in desiccation tolerant lichens. Besides the insights in the aforementioned DT mechanisms, additional protection strategies, never described before in lichens, have been reported. Some of these strategies are changes in the lipid membrane composition, the remodeling of the cell wall and changes in EPS, cytoplasm vitrification during desiccation, etc. Moreover, transcriptomic data analyses have provided clues about other possible components contributing to DT in lichens such as aquaporins or DPS. Another important insight is the beginning of the decryption of the molecular signaling pathways (NO, PLD, MAPKs, etc.) that are behind the rapid response of lichen phycobionts to dehydration.

**Figure 1:**
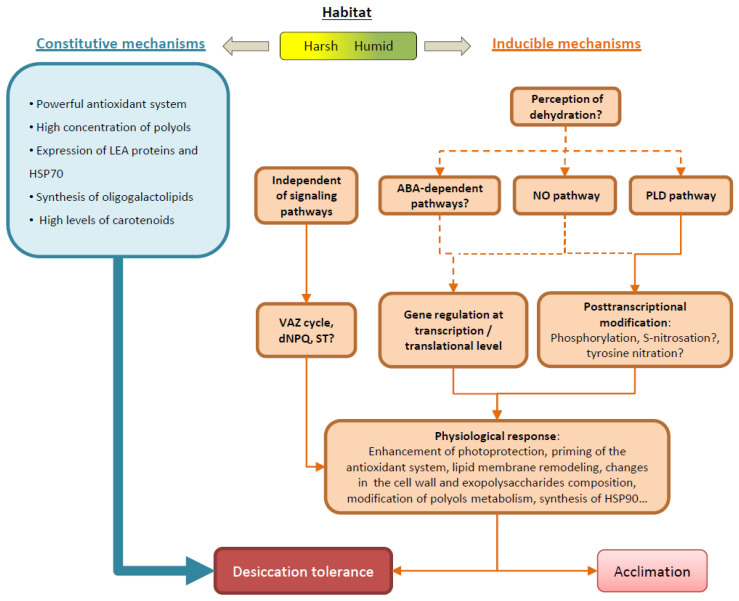
Lichen desiccation-tolerance (DT) is mainly achieved by constitutive mechanisms, although some physiological responses can also be induced during dehydration/rehydration. The relevance of constitutive and induced mechanisms may depend on adaption to habitat. In lichens growing in harsh habitats, DT relays mainly on constitutive mechanisms, whereas, in humid habitats, where drying occurs more slowly, lichens have enough time to induce cellular responses. The detection of cell water loss might trigger molecular signal cascades, such as the abscisic acid (ABA), nitric oxide (NO) and phospholipase D (PLD) pathways. Signaling pathways might regulate the expression of genes by the induction/inhibition of their transcription, or by the selective sequestration of specific mRNA that would be translated into proteins during rehydration. In addition, the activity of enzymes might be regulated by modifications such as phosphorylation. Besides regulation by signaling pathways, there are some physiochemical events indirectly caused by dehydration that can activate enzymatic reactions, such as the activation of the xanthophyll cycle (VAZ), the regulation of the photosynthetic state-transition (ST), or the activation of the desiccation non-photochemical quenching (dNPQ). The activation of these (and other) physiological responses would allow the cells to face a desiccation event in a better position, and to recover quickly upon rehydration. In this sense, priming of physiological responses could help lichens to acclimate to environmental conditions. For more detailed information see the text. Solid arrows depict known pathways, while dashed arrows indicate proposed or unknown pathways.

The compilation of previous and recent knowledge gives a clearer and detailed view of the molecular processes involved in the tolerance to desiccation of lichens (Figure 1). Desiccation-tolerance in lichens is mainly achieved by constitutive mechanisms, as it can be expected for poikilohydric organisms adapted to rapid fluctuation of hydration. However, there is increasing evidence that inducible responses during dehydration are also important. Inducible mechanisms would be particularly important for species growing in more humid and stable habitats, where constitutive mechanisms would have an unnecessary permanent energetic cost. Moreover, inducible factors could eventually contribute to acclimation and priming of physiological responses for a better adaption to changing environmental conditions. Several molecular signaling pathways might be involved in the transduction of the perception of water loss into physiological responses. The regulation of the function of proteins and enzymes may occur at different levels: (1) transcriptionally, up- or down-regulating the transcription of specific genes in response to D/R; (2) translationally, sequestering and stabilizing selected mRNA during desiccation to be translated into proteins upon rehydration; (3) through the activation/deactivation of enzymes by molecular modifications.

Despite the advances obtained during the last decade, there are still great challenges that remain unsolved. Among them, little is known about some key proteins involved in DT, such as LEAs or HSPs. Their study might be addressed through the genetic modification of the symbionts, or by comparative approaches employing symbionts differing in DT. Obviously, it is necessary to start the survey of specific DT components revealed by transcriptomic data analyses such as the aquaporins or DPRs. Another important challenge is to determine the DT degree of the different species/groups of phycobionts, and whether their selection might be related to the habitat. Finally, as the readers would have realised, the vast majority of DT studies have focused on lichen photobionts, through the analysis of particular parameters such as photosynthesis and plant pigments in lichen thalli, or directly working with in vitro algae cultures. The lack of specific studies with isolated mycobionts probably is due to the difficulty of their culture and manipulation, in comparison to microalgae. However, it is essential to begin the research in mycobionts since the fungus partner represents more than 90% of the biomass of a lichen thallus. The success of these goals will lead us to reach new horizons of knowledge in desiccation tolerance.

## Data Availability

Not applicable.
